# PD1/PD-L1 Axis Blockers in Head and Neck Squamous Cell Carcinoma: A Systematic Review and Meta-Analysis of Efficacy and Safety Outcomes

**DOI:** 10.7759/cureus.93579

**Published:** 2025-09-30

**Authors:** Tarang Patel, Sagar Dholariya, Siddhartha Dutta, Gyanendra Singh, Krupal Joshi, Ashwini Agarwal, Sanjay Gupta, Deepa Shukla

**Affiliations:** 1 Pathology, All India Institute of Medical Sciences, Rajkot, Rajkot, IND; 2 Biochemistry, All India Institute of Medical Sciences, Rajkot, Rajkot, IND; 3 Pharmacology, All India Institute of Medical Sciences, Rajkot, Rajkot, IND; 4 Community and Family Medicine, All India Institute of Medical Sciences, Rajkot, Rajkot, IND; 5 Microbiology, All India Institute of Medical Sciences, Rajkot, Rajkot, IND; 6 Forensic Medicine and Toxicology, All India Institute of Medical Sciences, Rajkot, Rajkot, IND; 7 Yoga (AYUSH), All India Institute of Medical Sciences, Jodhpur, Jodhpur, IND

**Keywords:** anti-pd1/pd-l1 inhibitors, head and neck squamous cell carcinoma (hnscc), meta-analysis, pd-l1 expression, systemic review

## Abstract

Cancers of the head and neck are common, and this review aimed to systematically analyze the safety and efficacy of anti-PD1/PD-L1 inhibitors (API) therapies in head and neck squamous cell carcinoma (HNSCC). This is a systematic review of randomized controlled trials (RCTs) showcasing the safety and efficacy of APIs in HNSCC. An online literature search of PubMed, Cochrane database, and ClinicalTrials.gov was conducted till May 31, 2024, to identify RCTs involving anti-PD1/PD-L1 therapies in oral cancer. The data were analyzed using Review Manager (RevMan) (Cochrane, London, United Kingdom), and the risk of bias was assessed for methodological quality. This study included nine RCTs with 3933 study participants. API treatment showed a significant improvement in overall survival (OS) compared to standard therapy in HNSCC (hazard ratio (HR) =0.85 (0.76,0.95), I^2^=26%, P=0.004). Even participants with PD-L1≥1%, treated with APIs, manifested substantial advancement of OS. Duration of response (DOR) was notably improved with API (HR= 0.31 (0.20, 0.50), I^2^=0%, P <0.00001) compared to standard therapy. Safety outcomes revealed comparatively reduced incidence of treatment-related adverse events and Grade 3/4 adverse effects when treated with APIs compared to standard therapy. In the era of emerging immunotherapy, APIs may be considered an alternative therapy in HNSCC, which could improve the OS, OS in patients with >1% PD-L1 expression, and DOR, as well as with a better safety profile as compared to chemotherapy and combination ICI. APIs pave the way for novel therapeutic strategies incorporating immune checkpoint inhibitors in HNSCC management.

## Introduction and background

Head and neck cancers, primarily head and neck squamous cell carcinoma (HNSCC), originate from diverse anatomic sites comprising the oral cavity mucosa and mucosa of other sites like nasopharynx, oropharynx, hypopharynx, and larynx. Cancers of the oral cavity and larynx have been typically linked to tobacco and/or excessive alcohol consumption. On the other hand, pharyngeal cancers are increasingly being linked to human papillomavirus (HPV) infection, namely HPV-16 [1,2].

Globally, head and neck cancers rank as the seventh most common cancer type, with approximately 888,000 new cases and 453,000 deaths reported in 2018 [3,4]. Asia has the highest proportion of global HNCs, accounting for 57.5% of cases. India has a significant burden of HNCs, with 30% of all cancer cases occurring there. In 2018, India reported 119,992 new oral cancer cases and 72,616 deaths, largely due to widespread tobacco use, which is implicated in 80-90% of oral cavity cancer cases [4-6]. The latest data from the Global Adult Tobacco Survey indicates that currently, 42.4% men, 14.2% women, and a combined total of 28.6% (266.8 million population) of the adult population consume tobacco in India [3,7].

Usually, less than 20% of patients with HNSCC have a clinical presentation of metastatic HNSCC [8]. However, 30-45% of those with locally advanced disease experience recurrence within a year. Traditionally, recurrent or metastatic HNSCC is treated with platinum-based chemotherapy combined with 5-fluorouracil, cetuximab, or taxanes like paclitaxel or docetaxel [9]. Nevertheless, this combination has been linked to substantial toxicity [10]. Following the administration of platinum and cetuximab, there are currently no validated therapy alternatives available. Methotrexate can still be used in these patients to achieve an overall survival (OS) of 6 months (median) and to have a 6% overall response rate [11,12].

Recently, there has been a growing recognition within the academic community of the importance of immune checkpoint inhibitors (ICI), particularly monoclonal antibodies targeting programmed cell death-1 (PD-1) and programmed cell death ligand-1 (PD-L1), in treating recurrent/metastatic HNSCC [13]. PD-1 (CD279) acts on T cells as an inhibitory receptor and is present on several immune cells, such as tumour-infiltrating CD8+ and CD4+ T cells, monocytes, B cells, dendritic cells, and NK (natural killer) cells. The PD-1 functions as an immunosuppressor by attaching to its ligands and helping to maintain immunological equilibrium [14]. PD-1 is known to bind to two different ligands, namely PD-L1 (B7-H1) and PD-L2. Various cells present with PD-L1 on their surface, like B cells and T cells, macrophages, squamous cells, vascular endothelial cells, and other tumour cells [14-16]. Tumour cells explore the PD-1/PD-L1 pathway to evade monitoring by immune cells and promote the growth of tumors [17]. Immunohistochemistry (IHC) is used to quantify PD-L1 levels, which helps predict the effectiveness of therapy against it [18].

In HNSCC, PD-L1 scoring on IHC is assessed using two primary methods: (i) Tumor Proportion Score (TPS) and (ii) Combined Positive Score (CPS). TPS is calculated as the percentage of PD-L1-positive tumor cells relative to the total viable tumor cells ×100. CPS includes the total number of PD-L1-positive tumor cells and inflammatory cells relative to the total viable tumor cells ×100. Another scoring method, the Immune Cell (IC) score, quantifies PD-L1 expression as the percentage of positive immune cells relative to the total tumor cells. The 22C3 and SP263 clones are used for TPS and CPS, with CPS using the 22C3 clone as the clinical standard, while the SP142 clone is used for IC scoring [19,20].

Recent studies have shown that anti-PD1/PDL1 inhibitors are highly efficacious in treating HNSCC, with significant efficacy and commendable safety data. Nivolumab and pembrolizumab, PD-1 inhibitors, increase overall survival in recurrent or metastatic (R/M) HNSCC cases that worsen with platinum-related therapy. Pembrolizumab used alone and in combination with chemotherapy has also shown effectiveness in treating tumors as initial therapies [10,12,21].

Given the limited literature, this systematic review and meta-analysis of nine carefully selected randomized controlled trials (RCTs) was performed to evaluate the pooled efficacy and safety of anti-PD-1/PD-L1 monotherapy in recurrent/metastatic HNSCC compared with standard therapy.

## Review

Materials and methods

Literature Search Planning

The present review was conducted following a predetermined methodology registered with the International Prospective Register of Systematic Reviews (PROSPERO) (registration number: CRD42024533601). It followed the Preferred Reporting Items for Systematic Reviews and Meta-Analyses (PRISMA) guidelines [22]. An ethical exemption certificate was obtained from the research cell of All India Institute of Medical Sciences, Rajkot, India (vide letter no IEC/AR/EFR/15/2024).

Literature search was done in the PubMed library, ClinicalTrials.gov, and Cochrane database, and data were screened till May 31, 2024. The Cochrane Handbook guideline was referred to for conducting this study. The MeSH (Medical Subject Headings) terms were used along with their corresponding entry terminology: “(pembrolizumab OR nivolumab OR durvalumab OR atezolizumab OR avelumab OR cemiplimab OR anti-PD1 OR anti-PDL1)” AND “(head and neck)” AND “(chemotherapy OR standard therapy)” AND “(recurrent OR metastatic)”. Furthermore, we conducted a manual literature search with the help of the articles’ references. We also utilized the Clinical Key search engine to obtain some full articles with results.

Selection Criteria

The RCTs were included after verifying the following eligibility criteria.

Inclusion criteria: (a) Diagnosed with head and neck squamous cell carcinoma, including mucosa of oral cavity, larynx, or pharynx, (b) RCTs, and (c) Anti-PD1/PD-L1 inhibitors as interventional therapies, while the other group may have received standard therapy and/or ICI combination therapy

Exclusion criteria: (a) Phase 1 RCT or non-RCTs, (b) Patients with SCC of nose, paranasal sinuses, or SCC of skin, (c) Experimental group receiving immune checkpoint inhibitors in addition to radiotherapy, (d) Study data not retrievable, (e) Study published in a language other than English, and (f) Animal studies.

The literature was evaluated by two separate authors utilizing a two-stage approach. Both authors thoroughly assessed the abstracts of all papers, pursued the inclusion/exclusion criteria, and selected research studies as per PRISMA 2020 recommendations.

Data Regarding Safety and Efficacy

The efficacy outcomes were measured by OS as a primary outcome to check the effectiveness of the drug. Secondary objectives were assessment of progression-free survival (PFS), objective response rate (ORR), and duration of response (DOR). The safety outcomes were assessed by treatment-related adverse events (TRAEs), serious adverse events (SAEs), Grade 3/4 adverse events, and treatment-related deaths.

The subgroup data analysis was conducted using PD-L1 expression data, including outcomes for patients having expression of PD-L1 ≥1% & PD-L1 ≥20%.

Data Extraction and Risk of Bias Evaluation

Two authors independently extracted the data, each adhering to acknowledged inclusion and exclusion criteria. The collected data included information like the name of author, ClinicalTrials.gov registry number, phase of RCT, trial arms, sample size, and the findings on effectiveness (OS with PD-L1 ≥1% and ≥20%, PFS, PFS with PD-L1 ≥1% and ≥20%, ORR, ORR with PD-L1 ≥ 1% and ≥ 20%, DOR, DOR with PD-L1 ≥1% and ≥20%) and safety. Two authors addressed and resolved discrepancies through discussion, involving other authors for additional input when necessary. The Cochrane Collaboration risk of bias 2 tool (ROB-2) was employed to assess the risk of bias, and the results were visualized using the RobVIS tool. This assessment considered bias arising from randomization processes, missing outcome data, deviations from intended interventions, selection of results, and outcome measurement. The ROB was categorized into three levels: low risk, high risk, and some concerns [23,24]. Two authors independently assessed the potential for bias and reached a consensus. When discrepancies persisted, they were fixed with the help of a third author or other contributing authors.

Data Integration and Analysis

Review Manager (RevMan) version 5.4 (2020; Cochrane, London, United States) was applied to conduct the pooled analysis using a random effect model.

The generic inverse variance method was applied to calculate the HR for OS and PFS, while ORR, DOR, and safety data were presented as dichotomous variables and were calculated using OR. The I² was assessed to calculate heterogeneity among studies [25]. The degree of inconsistency was evaluated using I^2^, and an I^2^ greater than 50% was taken as considerable heterogeneity [26]. Forrest plots were utilized to examine the study heterogeneity and to compare the control and experimental groups for given RCTs. p< 0.05 was determined to identify significance in statistics [27-30].

Results

Search Result

**Figure 1:**
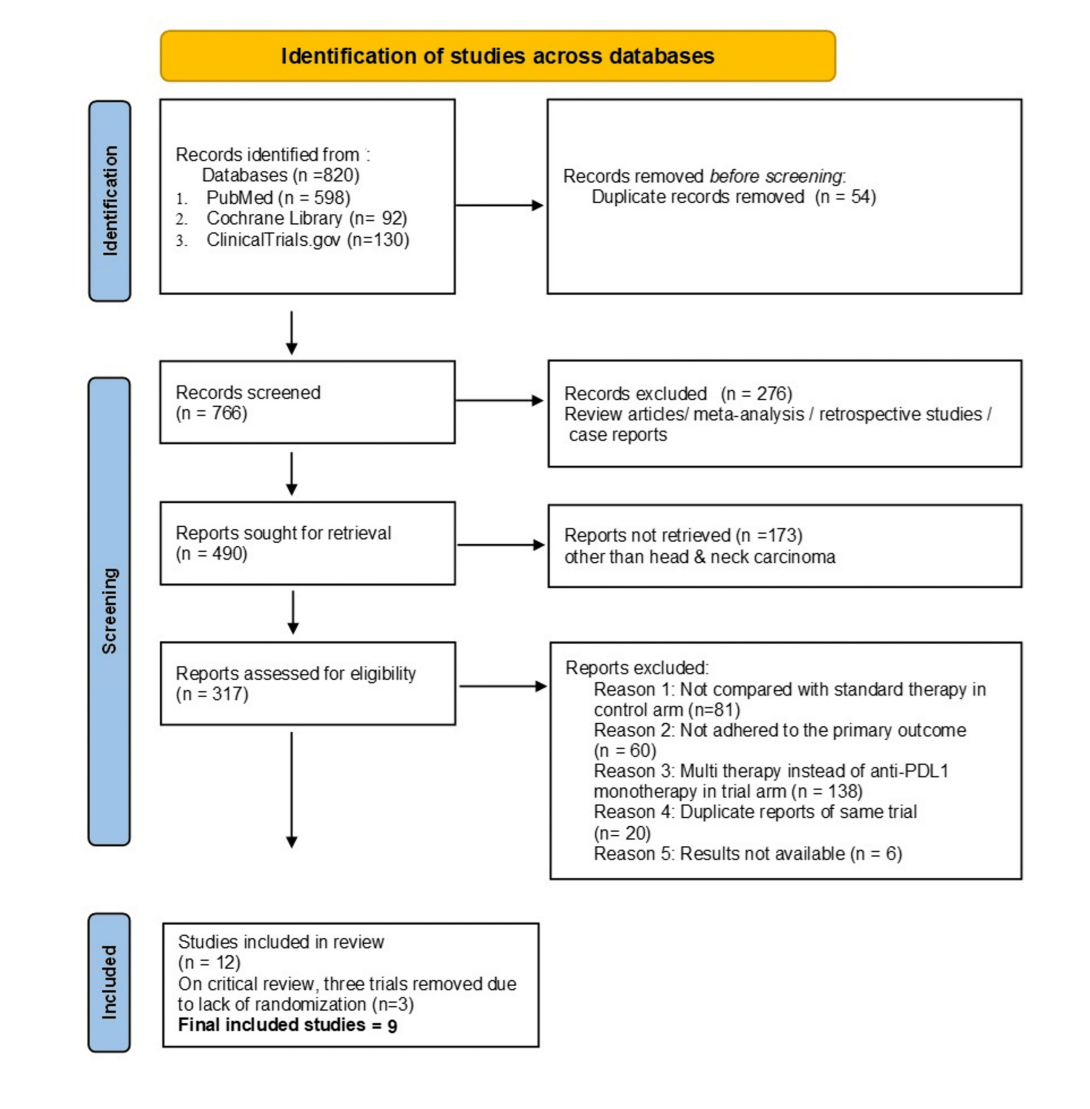
Systematic selection of articles as per PRISMA guidelines PRISMA: Preferred Reporting Items for Systematic Reviews and Meta-Analyses

A total of 820 studies were identified in the initial literature search. After a thorough screening process and excluding any duplicate articles, nine clinical trials were finally selected for the meta-analysis. The study selection was conducted as per the PRISMA 2020 guidelines and the details are depicted in Figure 1 [22].

The baseline characteristics, including study names, National Clinical Trial (NCT) number, RCT phase, sample size: total and arm-wise, age: overall and arm-wise, gender: overall and arm-wise, PD-L1 expression: overall and arm-wise, are mentioned in Table 1. 

**Table 1:** Baseline characteristics and the PD-L1 expression for HNSCC in included randomized controlled trials HNSCC: head and neck squamous cell carcinoma; NCT: National Clinical Trial; RCT: randomized controlled trial

Sr No	Author(s) Year	NCT number and RCT Phase	Trial Arm	Sample size, Total and arm wise	Age mean (standard deviation) Overall/Trial/Standard	Male and Female Overall/Trial/Standard	PD-L1 expression (TPS) Overall/Trial/Standard	PD-L1 expression (CPS) Overall/Trial/Standard
1	Psyrri et al., 2023 [31]	NCT02551159, Phase 3	Total	823	61.5	M-349, F-61	<25 : 282, ≥ 25 : 128	-
			Durvalumab	204	62.0 (26-89)	M-175 (85.8), F-29 (14.2)	<25 : 141 (69.1%), ≥ 25 : 63 (30.9%)	
			Durvalumab + Tremelimumab	413				
			Standard Treatment	206	61.0 (22-84)	M-174 (84.5), F-32 (15.5)	<25 : 141(68.4%), ≥ 25 : 65(31.6%)	
2	Harrington et al., 2023 [32]	NCT02358031, Phase 3	Total	601	61 (9.7)	M-735(83.3%), F-147(16.7%)	<50% : 683(77.4%), ≥ 50% : 199(22.6%)	<1 : 128 (14.5%), ≥ 1 : 754 (85.5%)
			Pembrolizumab monotherapy	301	61.2 (9.4)	M-250(83.1%), F 51(16.9%)	<50% : 234(77.7%), ≥ 50% : 67 (22.3%)	<1 : 44 (14.6%), ≥ 1 : 257 (85.4%)
			Standard Treatment	300	61.0 (10.0)	M-261(87%), F-39(13%)	<50% : 234 (78%), ≥ 50% : 66 (22%)	<1 :45 (15%), ≥ 1 :255 (85%)
3	Harrington et al., 2023 [33]	NCT02823574, Phase 2	Total	425			Overall <1: 128/320, ≥ 1: 192/320	-
			Nivolumab + Ipilimumab (ICI combination)	282				
			Nivolumab + Placebo (ICI monotherapy)	143				
			Combination vs monotherapy in platinum refractory group	241	59(24-82)	M-194 (80.5%) & F-47		
			Combination vs monotherapy in platinum eligible group	184	62(33-88)	M-152(82.6%) & F-32		
4	Okamoto et al., 2022 [34]	UMIN000031324, Phase 2	Nivolumab	22	63 (42-74)	M-20 (90.9%) F- 2 (9.1%)	Overall <1: 5/22, ≥ 1: 16/22, Not performed -1/22	-
5	Leddon et al., 2022 [35]	NCT03355560, Phase 2	Nivolumab salvage surgery	39	68 (49-85)	M-69% F-31%	-	Overall <1 : 6/34 (28%), ≥ 1 : 28/34 (72%)
6	Ferris et al. 2020 [36]	NCT02369874, Phase 3	Total	736	60	M-409, F-80	<25 : 349, ≥ 25 : 140	-
			Durvalumab	240	59.0 (24-84)	M-202/240(84.2%), F-38/240	<25 :172(71.7%), ≥ 25 :68(28.3%)	
			Durvalumab + Tremelimumab	247				
			Standard Treatment	249	61.0 (228-2)	M-207/249(83.1%), F-42/249	<25: 177(71.1%), ≥ 25: 72(28.9%)	
7	Ferris et al., 2016 [37]	NCT02105636, Phase 3	Total	361	59.1 (10.43)	M-300(83.1%), F-61 (16.9%)	-	<1 : 111, ≥ 1 :149, Not tested : 101
			Nivolumab	240	59.0 (10.15)	M-197(82.1%), F-43 (17.9%)		<1:73 (30.4%), ≥ 1:88 (36.7%), Not tested : 79(32.9%)
			Standard Treatment	121	59.4 (11.00)	M-103(85.1%), F-18 (14.9%)		<1:38(31.4%), ≥ 1:61 (50.4%), Not tested :22 (18.2%)
8	Schoenfeld et al., 2020 [38]	NCT02919683, Phase 2	Total	30			Overall <1: 5/25 (20%) ≥ 1: 20/25(80%)	
			Nivolumab + Ipilimumab (ICI combination)	15				
			Nivolumab only	15	62 (12)	M-18 (60%), F-12(40%)		
9	Cohen et al., 2019 [39]	NCT02252042, Phase 3	Total	495	60.2 (9.2)	M-412(83.2%),F-83(16.8%)	0% : 196 (39.6), 1-<50% :166 (33.5%) ≥ 50% :129 (26.1%), Missing: 04 (0.8%)	<1: 104 (21%), ≥ 1: 387 (78.2%), Missing :04 (0.8%)
			Pembrolizumab	247	60.3 (9.8)	M-207(83.3%), F-40(16.2%)	0% :103 (41.7), 1-<50% : 79 (32.0%), ≥ 50%: 64 (25.9%), Missing : 01 (0.4%)	<1: 50 (20.2%), ≥ 1: 196 (79.4%), Missing : 01 (0.4%)
			Standard Treatment	248	60.2 (8.6)	M-205(82.7%), F-43(17.3%)	0% : 93 (37.5), 1-<50%: 87 (35.1%), ≥ 50% : 65 (26.2%), Missing : 03 (1.2%)	<1 : 54 (21.8%), ≥ 1 : 191 (77%), Missing :03 (1.2%)
10	Zandberg et al., 2019 [40]	NCT02207530, Phase 2	Durvalumab	112	60(24-84)	M-80(71.4%), F-32(28.6%)	≥ 25: 112/112 (100%)	-
11	Siu et al., 2019 [41]	NCT02319044, Phase 2	Tremelimumab + Durvalumab	133			Zero (All cases were PD-L1 negative)	-
			Durvalumab	67	61 (42-77)	M-53 (79.1%), F-14 (20.9%)		
			Tremelimumab	67				
12	Cohen et al., 2018 [42]	NCT03358472, Phase 3	Total	89	62.5 (9.4)	M-45, F-9	-	-
			Pembrolizumab	19	63.0 (9.6)	M-16 (84.2), F-3 (15.8)		
			Pembrolizumab + Epacadostat	35				
			Standard Treatment	35	62.7 (10.0)	M-29 (82.9), F-6 (17.1)		

The efficacy results in the form of OS, OS in cases with PD-L1≥1%, OS in cases with PD-L1≥20% and also results of PFS, ORR, and DOR, along with PD-L1-related results, are presented in Table 2. 

**Table 2:** Efficacy results for HNSCC in included randomized controlled trials NM: not mentioned; NA: not available; HNSCC: head and neck squamous cell carcinoma; NCT: National Clinical Trial; RCT: randomized controlled trial

S. No	Author(s), Year	NCT no, and RCT Phase	Trial Arm	Sample size, total and arm wise	OS (months), median (95% CI)	OS with PD-L1 ≥1% (months), median (95% CI)	OS with PD-L1 ≥20% or high expression (months), median (95% CI)	PFS (months), median (95% CI)	PFS with PD-L1 ≥1%, (months), median (95% CI)	PFS with PD-L1 ≥20% or high expression (months), median (95% CI)	ORR, percentage (95% CI)	ORR with PD-L1 ≥1%, percentage (95% CI)	ORR with PD-L1 ≥20% or high expression, percentage (95% CI)	DOR (months), median (95% CI)	DOR with PD-L1 ≥1% (months), median (95% CI)	DOR with PD-L1 ≥20% or high expression (months), median (95% CI)
1	Psyrri et al., 2023 [31]	NCT02551159 Phase 3	Total	823			PD-L1 ≥50%,			PD-L1 ≥50%,			PD-L1 ≥ 50%,			PD-L1 ≥50%,
			Durvalumab	204	9.9 (8.9-11.9)		10.9 (9.0-14.3)	2.8 (2.0-2.8)		2.8 (1.7-4.2)	17.2 % (35/204)		16.2 % (16/99)	11.9 (4.6-17.8)		12.3 (5.6-18)
			Durvalumab + Tremelimumab	413	10.7 (9.6 -12.2)		11.2 (9.5-13.9)	2.8 (2.6 -2.9)		2.8 (2.6 -3.3)	21.8% (90/413)		25.3 %	9.2 (6.0 -19.6)		6.5(4.5 to 16.1)
			Standard Treatment	206	10.3 (9.0-12.1)		10.9 (8.3-13.4)	5.4 (4.4-5.7)		5.3 (4.3-5.8)	49.0% (101/206)		50.0 % (47/94)	4.2 (3.7- 4.5)		4.2 (3.0-5.7)
2	Harrington et al., 2023 [32]	NCT02358031 Phase 3	Total	601										NM	NM	NM
			Pembrolizumab monotherapy	301	11.5 (10.3 to 13.4)	12.3 (10.8 to 14.3)	14.8 (11.5 to 20.6)	2.3 (2.2 to 3.3)	3.2 (2.2 to 3.4)	3.4(3.2 to 3.8)	16.9 (12.9 to 21.7) (51/301)	19.1 (14.5 to 24.4) (49/257)	23.3 (16.4 to 31.4)			
			Standard Treatment	300	10.7 (9.3 to 11.7)	10.3 (9.0 to 11.5)	10.7 (8.8 to 12.8)	5.2 (4.9 to 6.1)	5.0 (4.8 to 6.0)	5.3(4.8 to 6.3)	36.0 (30.6 to 41.7) (108/300)	34.9 (29.1 to 41.1) (89/255)	36.1 (27.6 to 45.3)			
3	Harrington et al., 2023 [33]	NCT02823574 Phase 2	Total	425		NM(Not mentioned)	NM	NM	NM	NM	NM	NM	NM	NM	NM	NM
			Nivolumab + Ipilimumab (ICI combination)	282	9.76 (7.5 to 11.5)									NA (11.01-NA) 11.07 (4.14-NA)		
			Nivolumab + Placebo (ICI monotherapy)	143	11.30 (8.5 to 14.0)									27.04 (11.01-NA) 24.61 (6.90-NA)		
			Combination vs monotherapy in platinum refractory group	241	9.76 (6.51-11.37) vs 9.59 (7.13-14.26)			2.50 (1.45-2.76) vs 2.60 (1.54 -3.38)			13.2 (8.4-19.5) 18.3 (10.6 -28.4)					
			Combination vs monotherapy in platinum eligible group	184	9.71 (7.43-12.62) vs 12.91 (9.33-22.01)			2.76 (1.64-4.17) vs 2.86 (1.51 -5.65)			20.3 (13.6-28.5) 29.5 (18.5 -42.6)					
4	Okamoto et al., 2022 [34]	UMIN000031324 Phase 2	Nivolumab	22	17.4 months (9.1 to NC)	NM	NM	9.6 months (2.8 to NC)	NM	NM	36%	NM	NM	NM	NM	NM
5	Leddon et al., 2022 [35]	NCT03355560 Phase 2	Nivolumab salvage surgery	39	77.7% (64.1-94.2)	PD-L1 score and OS HR=1.0 (95% CI 0.97-1.03, p=0.9781	NM	71.4% (57.8-88.1%).	PD-L1 score and DFS HR=1.01 (0.99-1.3, p=0.275)	NM	NM	NM	NM	NM	NM	NM
6	Ferris et al., 2020 [36]	NCT02369874 Phase 3	Total	736		NM	,		NM	NM		NM	NM		NM	NM
			Durvalumab	240	7.6 (6.1-9.8)		9.8 (4.3-14.1)	2.1 (1.9-3.0)			17.9 (13.3–23.3) (43/240)			12.9 (6.9–21.0)		
			Durvalumab + Tremelimumab	247	6.5 (5.5-8.2)		4.8 (3.3 -6.4)	2.0 (1.9-2.3)			18.2 (13.6-23.6)			7.4 (2.8 to NA)		
			Standard Treatment	249	8.3 (7.3-9.2)		9.0 (6.8-11.0)	3.7 (3.1-3.7)			17.3 (12.8–22.5) (43/249)			3.7 (1.9–5.5)		
7	Ferris et al., 2016 [37]	NCT02105636 Phase 3	Total	361		NM	NM		NM	NM		NM	NM	NM	NM	NM
			Nivolumab	240	7.49 (5.49 to 9.10)			2.04 (1.91 to 2.14)			13.3 (9.3 to 18.3) (32/240)					
			Standard Treatment	121	5.06 (4.04 to 6.05)			2.33 (1.94 to 3.06)			5.8 (2.4 to 11.6) (7/121)					
8	Schoenfeld et al., 2020 [38]	NCT02919683 Phase 2	Total	30	89 % (78.3 to 10)	NM	NM	85% (72.4 to 99.7)	NM	NM		NM	NM	NM	NM	NM
			Nivolumab + Ipilimumab (ICI combination)	15							38 % (5/13)					
			Nivolumab only	15							13% (2/14)					
9	Cohen et al., 2019 [39]	NCT02252042, Phase 3	Total	495			NM			NM			NM			NM
			Pembrolizumab	247	8.4 (6.4 to 9.4)	8.7 (6.9 to 11.4)		2.1 (2.1 to 2.3)	2.2 (2.1 to 3.0)		14.6 (10.4 to 19.6) (36/247)	17.3 (12.3 to 23.4) (42/247)		18.4 (2.7 to 18.4)	18.4 (2.7 to 18.4)	
			Standard Treatment	248	6.9 (5.9 to 8.0)	7.1 (5.7 to 8.3)		2.3 (2.1 to 2.8)	2.3 (2.1 to 3.0)		10.1 (6.6 to 14.5) (25/248)	9.9 (6.1 to 15.1) (24/248)		5.0 (1.4 to 18.8)	9.6 (1.4 to 18.8)	
10	Zandberg et al., 2019 [40]	NCT02207530 Phase 2	Durvalumab	112	7.1 (4.9-9.9)	NM	NM	2.1 (1.9-3.7)	NM	NM	16.2% (9.9-24.4)	NM	NM	NM	NM	NM
11	Siu et al., 2019 [41]	NCT02319044 Phase 2	Tremelimumab + Durvalumab	133	7.6 (4.9-10.6)	NM	NM	2.0 (1.9-2.1)	NM	NM	7.8%(10/129) [3.78-13.79]	NM	NM	NM	NM	NM
			Durvalumab	67	6.0 (4.0-11.3)			1.9 (1.8-2.8)			9.2% (6/65) [3.46-19.02]					
			Tremelimumab	67	5.5 (3.9-7.0)			1.9 (1.8-2.0)			1 (1.6) [0.04-8.53]					
12	Cohen et al., 2018 [42]	NCT03358472 Phase 3	Total	89	NM	NM	NM	NM	NM	NM	-	NM	NM	NM	NM	NM
			Pembrolizumab	19							21.1 % (6.1- 45.6) (4/19)					
			Pembrolizumab + Epacadostat	35							31.4 (16.9-49.3)					
			Standard Treatment	35							34.3 % (19.1-52.2) (12/35)					

The safety results regarding TRAEs, SAEs, and treatment-related deaths are included in Table 3. 

**Table 3:** Safety results for HNSCC in included randomized controlled trials NM: not mentioned; NCT: National Clinical Trial; RCT: randomized controlled trial; TRAE: treatment-related adverse event; SAE: serious adverse event; ICI: immune checkpoint inhibitor

Sr No	Author(s), Year	NCT Number, and RCT Phase	Trial Arm	Sample Size	TRAE	SAE	Grade 3 or 4 AE	Treatment-Related Death
1	Psyrri et al., 2023 [31]	NCT02551159 Phase 3	Total	823		NM		
			Durvalumab	204	92/204		18/204	4/202
			Standard Treatment	206	184/206		104/196	2/196
2	Harrington et al., 2023 [32]	NCT02358031 Phase 3	Total	601				
			Pembrolizumab	301	175/300	123/300	51/300	2/300
			Standard Treatment	300	278/300	141/287	199/287	2/287
3	Harrington et al., 2023 [33]	NCT02823574 Phase 2	Total	425			75/423	
			Nivolumab + Ipilimumab (ICI combination)	282	179/280	31/280	55/280	0/280
			Nivolumab + Placebo (ICI monotherapy)	143	95/143	11/143	20/143	0/143
4	Okamoto et al., 2022 [34]	UMIN000031324 Phase 2	Nivolumab	22	12 /22	NM	4/22	0/22
5	Leddon et al., 2022 [35]	NCT03355560 Phase 2	Nivolumab salvage surgery	NM	31/39	NM	3/39	0/39
6	Ferris et al., 2020 [36]	NCT02369874 Phase 3	Total	736				
			Durvalumab	240	136/237	17/237	24/237	4/237
			Durvalumab + Tremelimumab	247	150/246	29/246	40/246	2/246
			Standard Treatment	249	197/240	15/240	58/240	0/240
7	Ferris et al., 2016 [37]	NCT02105636 Phase 3	Total	361				
			Nivolumab	240	139/236	165/236	31/236	1/236
			Standard Treatment	121	86/111	87/111	39/111	0/111
8	Schoenfeld et al., 2020 [38]	NCT02919683 Phase 2	Total	30				
			Nivolumab + Ipilimumab (ICI combination)	15	12/15	2/15	5/15	2/15
			Nivolumab only	15	11/15	3/15	2/15	2/15
9	Cohen et al., 2019 [39]	NCT02252042, Phase 3	Total	495				
			Pembrolizumab	247	155/246	110/246	33/246	5/246
			Standard Treatment	248	196/234	92/234	85/234	4/234
10	Zandberg et al., 2019 [40] [36]	NCT02207530 Phase II	Durvalumab	112	64/112	43/112	9/112	0/112
11	Siu et al., 2019 [41]	NCT02319044 Phase 2	Tremelimumab + Durvalumab	133	110/133	59/133	21 (15.8)	1 of 133
			Durvalumab	67	52/65	18/65	8 (12.3 %)	0 of 67
12	Cohen et al., 2018 [42]	NCT03358472 Phase 3	Total	89			NM	NM
			Pembrolizumab	19	17/19	8/19		
			Pembrolizumab + Epacadostat	35	34/34	12/34		
			Standard Treatment	35	34/34	12/34		

**Figure 2:**
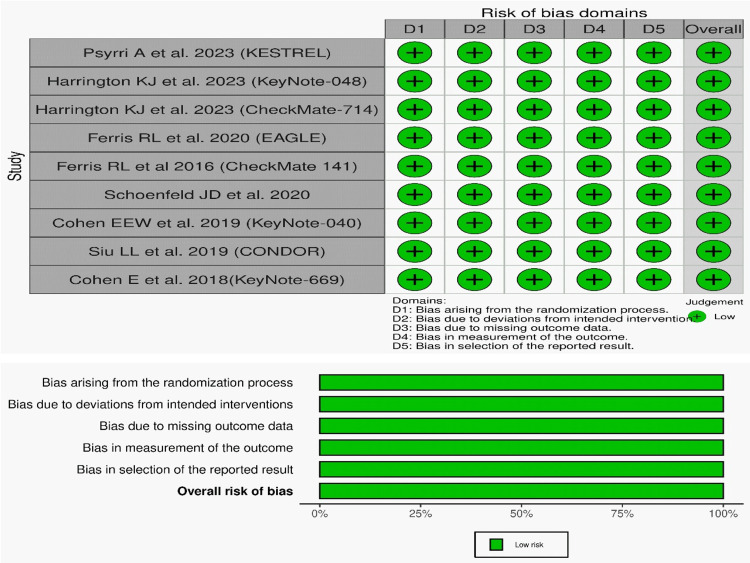
Risk of bias of included studies References: [31-33,36-39,41,42].

From the selected nine RCTs, a total of 3933 patients in all were analyzed. Among them, six were phase 3 RCTs and three were phase 2 RCTs. All studies examined the safety and efficacy of API monotherapy in at least one arm (Table 2, 3). All nine studies examined the efficacy of the APIs. OS was studied by eight RCTs. OS was compared for API monotherapy and standard therapy in five RCTs. OS of PD-L1≥1% cases was examined by two RCTs, whereas OS of PD-L1≥20% was examined by three RCTs. PFS was examined by eight RCTs. PFS of API monotherapy and standard therapy was studied by five RCTs. PFS of API monotherapy and ICI combination therapy was also studied by three RCTs. PFS was also studied in association with PD-L1 expression. ORR was examined by eight RCTs. ORR was studied between API monotherapy and standard therapy in six cases. PD-L1 positivity was also explored. DOR was inquired by three RCTs. Data related to TRAE, SAE, grade 3 or 4 adverse effects, and death events probed by various RCTs [31-42]. All RCTs showed a minimal risk of bias based on the RoB 2. The overall conclusion of the ROB evaluation is depicted in Figure 2.

Assessment of Efficacy Results

**Figure 3:**
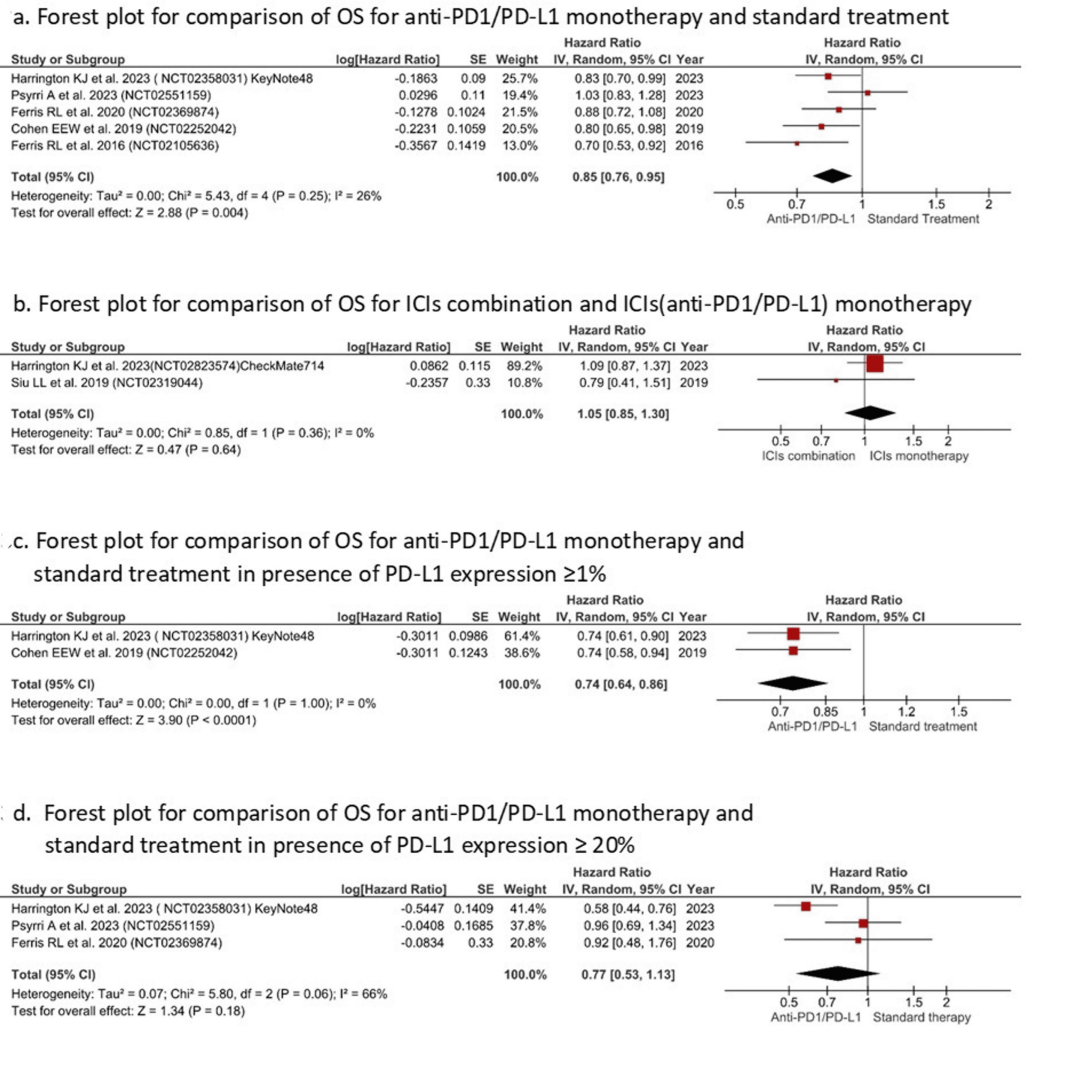
Various forest plot for comparison of overall survival (Y-axis) for API monotherapy with standard treatment/ICI combination therapy (X-axis) References [31-33,36,37,39,41]. ICI: immune checkpoint inhibitor; API: anti-PD1/PD-L1 inhibitor; OS: overall survival

OS: The pooled analysis of RCTs showed significant difference in OS, comparing API monotherapy with standard therapy, favouring API monotherapy (HR=0.85 (0.76, 0.95), I^2^= 26%, Z=2.88, P=0.004) (Figure 3a), but no significant difference was observed while comparing combination ICI therapy with API monotherapy (HR=1.05 (0.85, 1.30), I^2^=0%, p=0.64) (Figure 3b). Also, a significant improvement in OS (cases with PD-L1 ≥1%) was evident with API monotherapy compared with standard of care (SOC) (HR=O.74 (0.64, 0.86), I^2^= 0%, Z=3.90, P<0.0001), favoring API monotherapy (Figure 3c1). Nonetheless, the improvement was insignificant in cases having PD-L1≥20% (HR= 0.77(0.53, 1.13), I^2^=66%, P=0.18) (Figure 3d).

**Figure 4:**
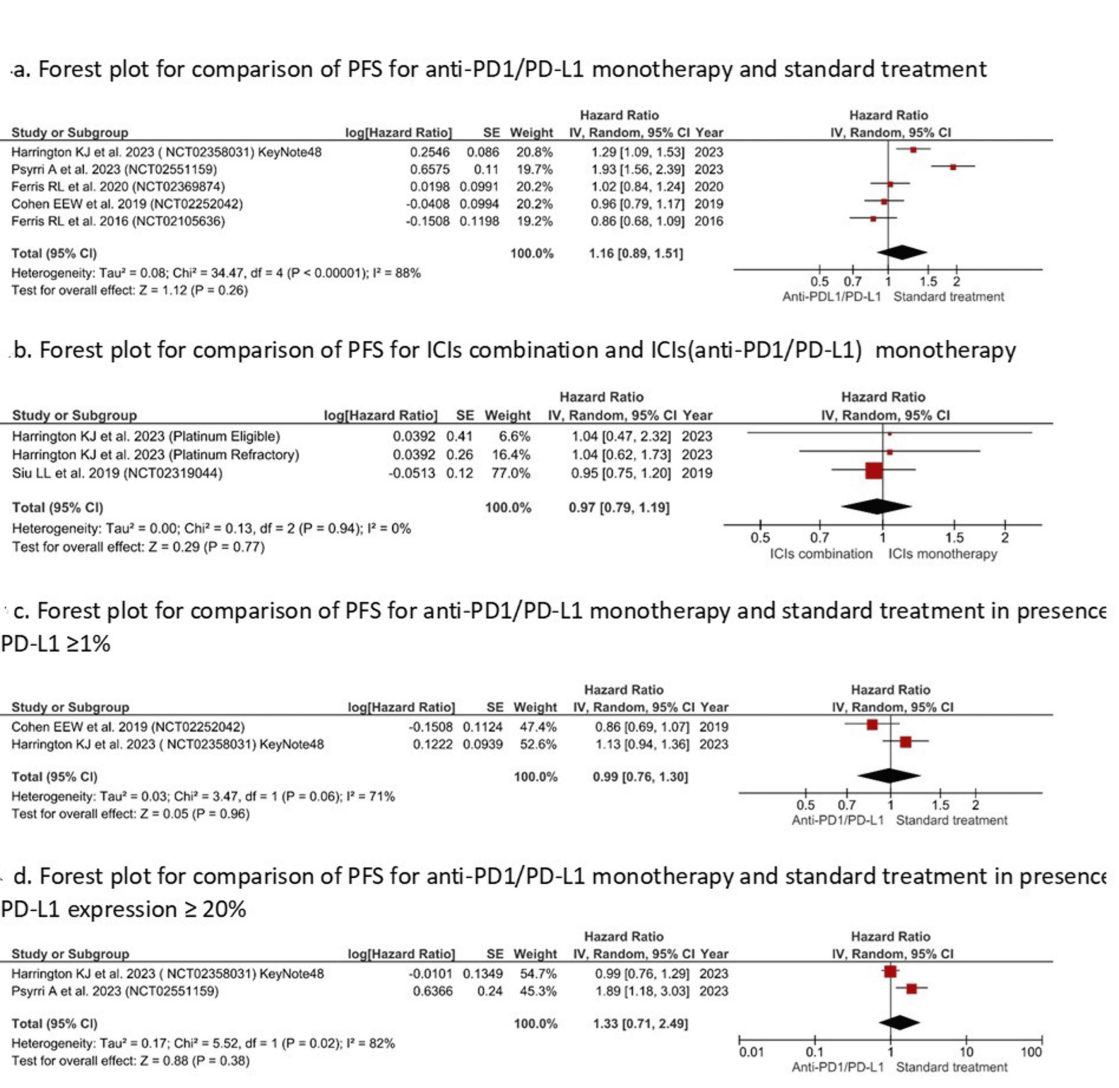
Forest plot for comparison of PFS (Y-axis) for API monotherapy with standard treatment/ICI combination therapy (X-axis) References [31-33,36,37,39,41]. ICI: immune checkpoint inhibitor; API: anti-PD1/PD-L1 inhibitor; PFS: progression-free survival

PFS: No significant difference was established in PFS using API monotherapy when compared with standard therapy (HR=1.16 (0.89, 1.51), I^2^=88%, P=0.26])m (Figure 4a) or compared with combination therapy (HR=0.97 (0.79, 1.19), I2=0%, P=0.77) [Figure 4b). Also, PFS difference was not significant between API monotherapy and standard therapy in cases of PD-L1 expression ≥1% (HR=0.99 (0.76, 1.30), I^2^=71%, P=0.96) (Figure 4c) and in cases of PD-L1 expression ≥20% (HR=1.33(0.71, 2.49), I^2^=82%, P=0.38) (Figure 4d).

**Figure 5:**
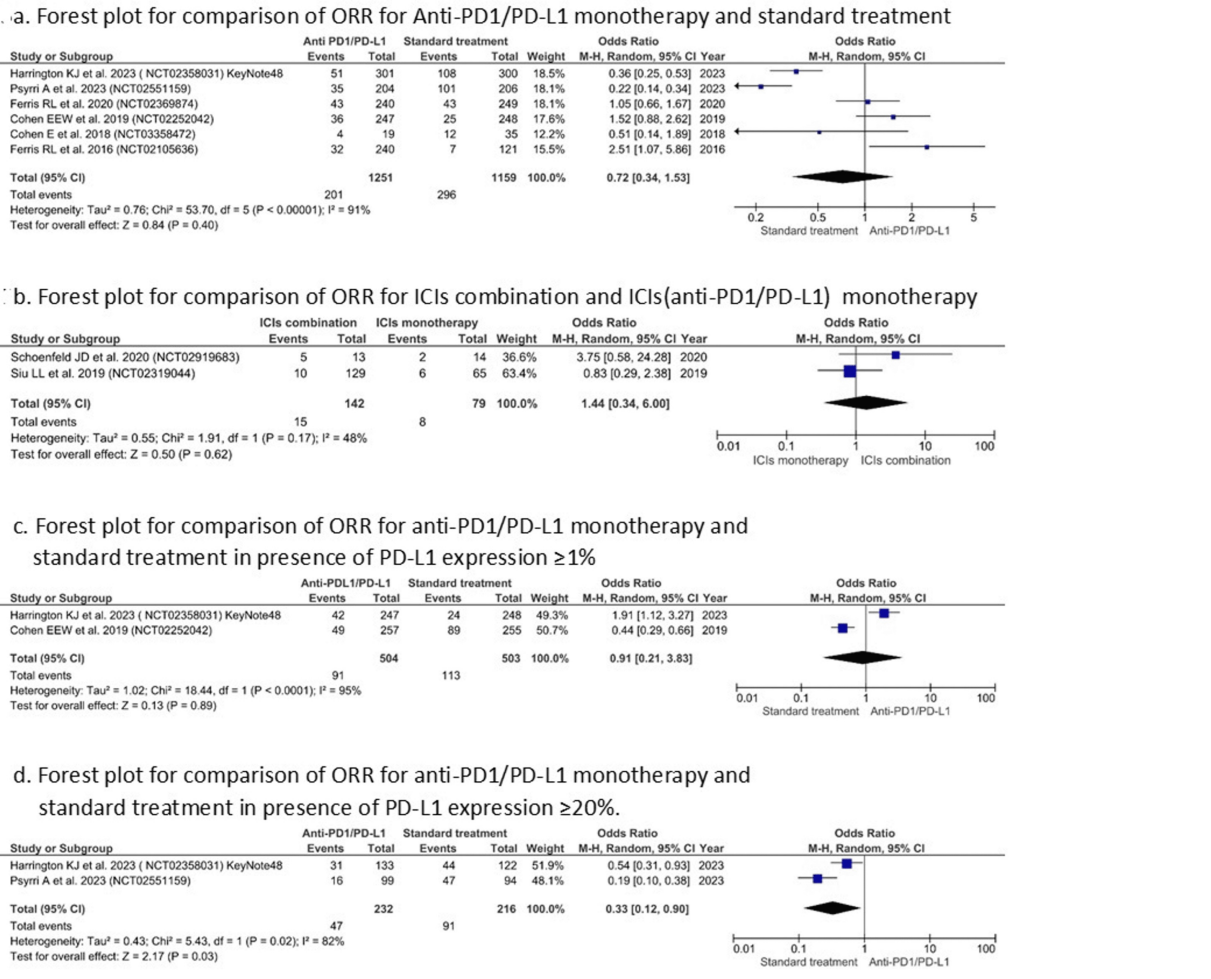
Forest plot for comparison of ORR (Y-axis) for API monotherapy with standard treatment/ICI combination therapy (X-axis) References [31,32,36-39,41,42]. ICI: immune checkpoint inhibitor; API: anti-PD1/PD-L1 inhibitor; ORR: objective response rate

ORR: While evaluating ORR, no significant difference was identified with API monotherapy when compared with either standard therapy (OR=0.72 (0.34, 1.53), I2=91%, P=0.40) (Figure 5a] or combination therapy (OR=1.44 (0.34, 6.0), I2=48%, p=0.62) (Figure 5b) Similar insignificant results were observed in cases with presence of PD-L1 expression ≥1% [OR= 0.91 (0.21, 3.83), I^2^=95%, p=0.89] (Figure 5c). However, cases with ≥20% PD-L1 levels indicated significant improvement in ORR in cases treated with standard therapy when compared to API monotherapy (OR= 0.33 (0.12, 0.90), I^2^=82%, p=0.03). The high heterogeneity likely arises from differences in trial design, patient selection criteria, and variation in comparator arms across the included studies. To account for this, we applied a random-effects model (Figure 5d).

**Figure 6:**
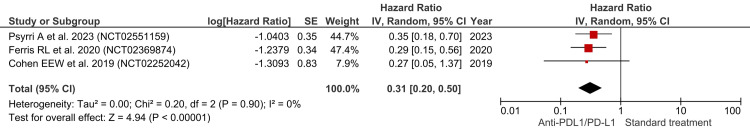
Forest plot for comparison of DOR (Y-axis) for API monotherapy and standard treatment (X-axis). References [31,36,39]. API: anti-PD1/PD-L1 inhibitor; DOR: duration of response

DOR: DOR expressed significant improvement in the API monotherapy arm compared to the standard therapy arm (HR= 0.31 (0.20, 0.50), I^2^=0%, P <0.00001) (Figure 6).

Assessment of Safety Outcomes

**Figure 7:**
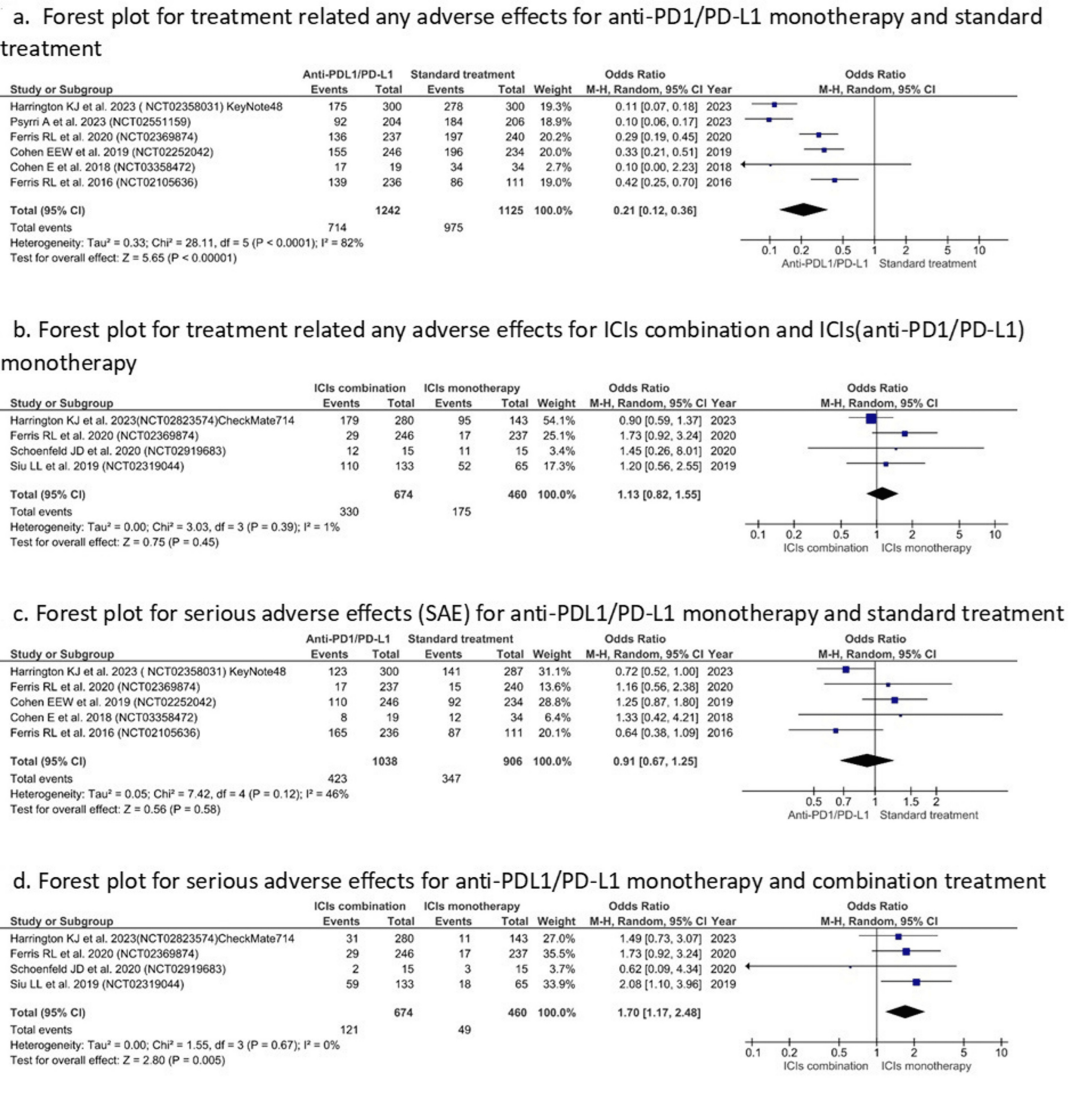
Forest plots for TRAE/SAE (Y-axis) for API monotherapy with standard treatment/ICI combination therapy (X-axis). References [31-33,36-39,41,42]. TRAE: treatment-related adverse event; SAE: serious adverse event; API: anti-PD1/PD-L1 inhibitor; ICI: immune checkpoint inhibitor

Treatment-related adverse effects were significantly lower for API monotherapy compared to standard treatment (OR = 0.21 (0.12, 0.36), I^2^=82%, Z=5.65, P <0.00001) (Figure 7a). Although no significant difference was revealed while comparing API monotherapy with combination therapy (OR = 1.13 (0.82, 1.55), I^2^=1%, P=0.45) (Figure 7b). Likewise, the difference was not significant in the case of SAEs while comparing API monotherapy with standard treatment (OR =0.91 (0.67, 1.25), I^2^=46%, P=0.58) (Figure 7c). However, SAEs were notably lower in API monotherapy while comparing with the ICI combination group (OR=1.70 (1.17, 2.48), I^2^=0%, Z=2.80, P=0.005) (Figure 7d).

**Figure 8:**
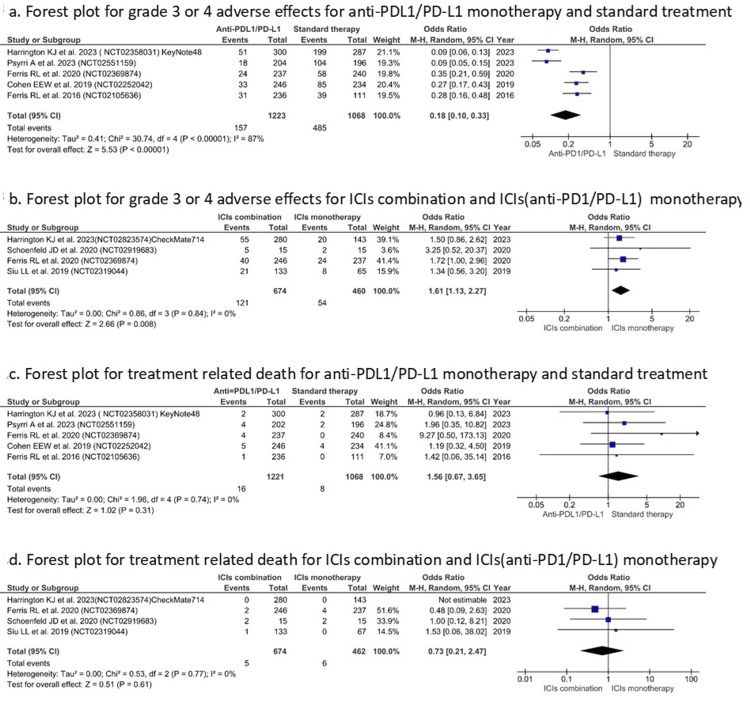
Forest plot for grade 3/4 adverse effects and treatment related death (Y-axis) for API monotherapy (X-axis). References [31-33,36-39,41]. API: anti-PD1/PD-L1 inhibitor

Grade 3/4 adverse events were significantly lower with API monotherapy, compared to standard treatment (OR=0.18 (0.10, 0.33), I^2^=87%, Z=5.53, P<0.00001) (Figure 8a); and also lower in comparison to ICI combination therapy (OR=1.61 (1.13, 2.27), I^2^=0%, Z=2.66, P=0.008) (Figure 8b). The difference was not significant in the case of therapy-related deaths for anti-PDL1/PD-L1 monotherapy when compared with both standard treatment (P=0.31) (Figure 8c) and combination therapy (P=0.61) (Figure 8d).

Discussion

HNSCC involves the mucosa of the oral cavity, larynx, pharynx, contributing to worldwide mortality. The chemotherapy drugs (platinum-based) are the contemporary drug of choice for cases of recurrent or metastatic HNSCC (R/M-HNSCC). However, they have demonstrated limited improvements in survival, with just 10-12 months of median OS [43,44]. The present meta-analysis observed that API monotherapy has potential in improving OS for inclusive cases of HNSCC and also for cases with PD-L1 expression of ≥1% along with a better safety profile as compared to the standard therapy. 

The early discontinuation of treatment or the emergence of resistance to treatment are the usual outcomes of chemotherapy treatment [39]. Therefore, it is necessary to explore alternative immunotherapy options for patients with HNSCC.

The PD1/PD-L1 pathway serves as a crucial immunological checkpoint that hinders the activation of T cells. It may be specifically addressed to revive the T cells' immune response against tumour cells [45]. There is a scarcity of published meta-analyses on the utilization of API immunotherapy in recurrent or metastatic HNSCC. One meta-analysis contained just two RCTs on PD1 inhibitors [46]. Another meta-analysis includes three RCTs that used API monotherapy in R/M-HNSCC cases [47]. A meta-analysis by Chen et al. included four cases of API in HNSCC [43]. Another study by Dang et al. included eight RCTs exhibiting the safety profile and effectiveness of various ICI, including API, in R/M-HNSCC cases [48]. With nine RCTs, the present study has pooled the existing evidence to provide an updated review on the use of just anti-PD1/PD-L1 immunotherapy in HNSCC patients.

The present meta-analysis initially included 12 RCTs. However, upon critical evaluation, three phase 2 trials were excluded due to their non-randomized design. Okomata et al. conducted a prospective, single-arm study using nivolumab in R/M-HNSCC without a comparator group [34]. Leddon et al. performed an open-label, single-arm, phase 2 clinical trial following salvage resection in recurrent HNSCC, without randomization [35]. Zandberg et al. conducted a non-randomized phase 2 trial using durvalumab monotherapy without a control arm [40]. Due to the absence of randomization and comparator groups, these studies were excluded to maintain the methodological rigor of the meta-analysis.

Recent research has shown that among cases of R/M-HNSCC, API monotherapy depicts anti-tumour effectiveness and safety. In patients of platinum-refractory R/M-HNSCC, Yen et al.'s study (Checkmate 141) showed a significant increase in OS and PFS with nivolumab usage [21]. Furthermore, it has been observed that nivolumab is associated with a decreased occurrence of high-grade toxicities (grade 3 or above) in comparison to the SOC, with rates of 13.1% and 35.1% respectively. Another study (KEYNOTE 040) highlighted that pembrolizumab presented with substantial betterment of OS in comparison to the investigator’s treatment of choice, which included methotrexate, docetaxel, or cetuximab [39]. Patients of R/M-HNSCC, with disease advancement during/after chemotherapy, demonstrated remarkable improvement. Furthermore, TRAEs associated with pembrolizumab were decreased when compared with the SOC. The KEYNOTE 048 study concluded that pembrolizumab, along with 5-fluorouracil and platinum, is a suitable preliminary therapy for R/M-HNSCC [32]. On the other hand, pembrolizumab used alone is a suitable initial treatment for cancer patients showing PD-L1 positivity on tissue expression. Overall findings indicate that comparing the first-line API monotherapy use with SOC is a promising and worthwhile proposal to consider for further investigation.

This meta-analysis found that using API treatment alone substantially improved OS in all cases of HNSCC (HR=0.85 (0.76, 0.95), I^2^= 26%, P=0.004) and also in cases having PD-L1 expression ≥1% (HR=O.74 (0.64, 0.86), I^2^= 0%, Z=3.90, P<0.0001); while compared to the participants treated with SOC. The results suggest that the level of PD-L1 expression may serve as an indicator for predicting the effectiveness of API monotherapy in patients with R/M-HNSCC. Results of another meta-analysis by Chen et al. [41] agree with the findings of our study. They expressed that API monotherapy remarkably improved OS for cases with ≥1% PD-L1 (HR = 0.67 (0.53, 0.81), p <0.001). However, improvement was insignificant in OS for cases having <1% PD-L1 levels (HR = 1.05, (0.63, 1.47)) [41].

Nevertheless, our meta-analysis revealed no significant improvement in OS in participants with ≥20% PD-L1 value (HR= 0.77(0.53, 1.13), I^2^=66%, P=0.18), while applying API monotherapy compared to SOC. The heterogeneity observed may be attributed, in part, to the distinct outcomes associated with anti-PD1 and anti-PDL1 medications. Furthermore, the assessment techniques for measuring the PD-L1 levels and cut-off levels differed among a few studies. This could perhaps be another factor contributing to the heterogeneity. The levels of PD-L1 in tumour cells are considered as a reasonable biological marker in cases of R/M HNSCC; however, it’s not free of any constraints.

Regarding the tolerability of the drugs, our study presented API monotherapy as having a superior safety profile in contrast to SOC. API monotherapy presented with reduced incidence of TRAEs, compared with SOC, and also with limited incidence of SAEs, compared with ICI combination therapy. In comparison to chemotherapy and/or combination ICI drugs therapy, there were reduced occurrences of grade 3/4 TRAEs with PD-1/PD-L1 inhibitor monotherapy. These findings matched with those of Chen et al. [43] and Mok et al. [49], respectively. The latter study found that pembrolizumab presented with reduced grade 3/4 TRAEs, when compared with chemotherapy (18% vs 41%), when used as the initial treatment in cases of non-small cell pulmonary cancer, either localised to the lung or metastatic. In R/M-HNSCC cases, accordingly, API immunotherapy demonstrated reasonable data regarding safety profile.

Masarwy et al. performed research to estimate the effectiveness and safety profile of API monotherapy in cases of surgically removable head and neck cancer [50]. They found that neoadjuvant API treatment was safely administered. The study reported a pathological response rate of 9.7% (95%CI, 3.1-18.9%) in the primary tumour sites, and there was a 2.9% complete response observed in the pathological analysis (95% CI, 0-9.5%). The results indicated that API have the potential to provide benefits not only for R/M-HNSCC cases but also for patients with surgically resectable HNSCC, showing encouraging anticancer effects.

The present meta-analysis has certain limitations. Firstly, among the nine RCTs included, three were phase 2 trials. These phase 2 studies primarily focused on assessing the efficacy and safety of the intervention but did not include a direct comparison with the SOC therapy. As a result, their findings may not provide definitive evidence on the superiority or non-inferiority of the intervention compared to established treatment protocols. Furthermore, these investigations encompassed diverse anti-PD1/PD-L1 regimens and distinct eligible populations, potentially leading to diversity in the meta-analysis. Also, there is heterogeneity in the anti-tumour actions by human immunity and the API mechanism of action, which can be attributed to their differences. Additionally, it is worth noting that none of the RCTs incorporated in the study offered either data on the survival rates of HPV-positive individuals or subgroup analyses on the p16 factor. Another limitation is the effect of radio-chemotherapy on PD-L1 tissue expression. Recent evidence suggests that marker expression increases (at a 1% cutoff) in patients undergoing radiochemotherapy with platinum-based drugs [51]. It is crucial to consider these factors in future research and evaluate their significance when possible. Although our meta-analysis was not designed to directly evaluate cost-effectiveness or patient-reported quality-of-life outcomes, these factors remain critical in determining the real-world applicability of API therapy and should be prioritized in future clinical studies.

## Conclusions

API monotherapy has shown potential in improving OS for inclusive cases of HNSCC and also for cases with PD-L1 expression of ≥1%, compared to the standard therapy. TRAEs were significantly lower with API monotherapy than with SOC. Even SAEs were lower among the API monotherapy arm compared to the ICI combination arm. Furthermore, Grade 3 or 4 TRAE incidence was lower with API monotherapy in comparison to both standard treatment and ICI combination therapy. However, API monotherapy did not show a significant difference in PFS or ORR in contrast to SOC or combination therapy, although DOR was significantly improved with API monotherapy compared to SOC. Therefore, our findings support the use of API monotherapy in cases of R/M-HNSCC showing levels of PD-L1≥1%. The cellular levels of PD-L1 markers might serve as a biological marker for recognising patients who may show a positive response with API monotherapy. There is a need to establish standardized criteria for evaluating the PD-L1 expression scenario in future clinical studies.
